# Chemicals regulation and non-animal methods: displacing the gold standard

**DOI:** 10.12688/wellcomeopenres.20581.2

**Published:** 2024-09-02

**Authors:** Annamaria Carusi

**Affiliations:** 1 Interchange Research, London, E5 8JW, UK

**Keywords:** non-animal methods, chemicals regulation, risk assessment, validation

## Abstract

Regulating industrial chemicals in foodstuffs and consumer products is a major aspect of protecting populations against health risks. Non-animal testing methods are an essential part of the radical change to the framework for toxicity testing that is long overdue in global economies. This paper discusses reasons why the drive to reduce animal testing for chemical safety testing is so difficult to achieve, as perceived by those who are closely involved in chemicals regulations in different capacities. Progress is slow, despite the fact that the ethico-legal conditions for a move away from animal testing are largely in place, and despite scientific arguments for a radical change in the paradigm of toxicity testing, away from reliance on animal studies. I present empirical data drawn from two studies in a European Commission context promoting non-animal methods. The aim of the paper is modest. It is to foreground the voices of those who deal with the science and regulation of chemicals on a day-to-day basis, rather than to offer a theoretical framework for what I heard from them. I offer a synthesis of the main challenges faced by non-animal alternatives, as these are perceived by people in different stakeholder groups dealing with chemicals regulation. I show where there are pockets of agreement between different stakeholders, and where the main disagreements lie. In particular there is dispute and disagreement over what counts as validation of these alternative tests, and by implication of the traditional ‘gold standard’ of animal testing. Finally, I suggest that the shift to non-animal methods in chemicals regulation demonstrates the need for the concept of validation to be broadened from a purely techno-scientific definition, and be more explictly understood as a demand for trust and acceptance, with more attention given to the complex social, institutional and economic settings in which it operates.

## Introduction

Chemical safety testing is a profoundly political technoscience, and is increasingly in the public eye. There is growing awareness of the consequences for health and the environment of the pervasiveness of industrial chemicals that are often little understood. After the scandals of sulfanilamide, DDT, asbestos and thalidomide, there are ongoing urgent questions about a multitude of food, household, consumer and agricultural products, not to speak of heavy industrial and military eqipment. Rachel Carson’s *Silent Spring* (1962), published in 1962, was for many a rude awakening into the realities of chemical exposure, and has been followed by a steady stream of books and interventions. Unsurprisingly, chemicals are attractors for a variety of interests that are more often than not in conflict: human health, environment, animal, industrial, governance and national and international political interests may all come into the mix of competing interests ( Boudia & Jas, 2013). Several studies have shown the extent to which industry interests are able to bias the scientific evidence concerning toxicity for regulation ( Henry *et al.*, 2021). Another way in which industry interests prevail is in the huge number of chemicals in industrial products of all kinds for which there is no information. A 1997 report by the Environmental Defense Fund, labelled as *Toxic Ignorance* the lack of knowledge of whether regularly used chemicals are safe or not ( EDF, 1997).

A further challenge comes from the need to reform the scientific paradigm of chemicals safety testing. On the one hand, there is legislative pressure from the 2010 EU Directive on the protection of animals in scientific research, which ‘[…] represents an important step towards achieving the final goal of full replacement of procedures on live animals for scientific and educational purposes as soon as it is scientifically possible to do so’ (Para 10) ^1^ To that end, it seeks to facilitate and promote the advancement of alternative approaches. The EU is only one global player in shaping scientific research, and there is a wide range of variation between nations and states. However, this shift is also predicated upon scientific and pragmatic considerations. Currently underway are deep shifts away from the use of animals as the gold standard for safety testing, and towards a wide array of non-animal methods. Overlapping with this shift but not entirely subsumed by it is the shift from whole organism approaches to mechanistic approaches. This is a monumental shift in chemicals safety testing, which wil warrant the title of paradigm shift; whether or at least the speed with which it will occur depends on an intricate interlacing of science with social, economic and institutional factors. This paper focuses on the questions, doubts and challenges of attempts to establish non-animal approaches as trustable indicators of the safety and risks of chemicals for health and environment, as these are perceived by scientists, regulators, and risk assessors working in the broad field of regulatory toxicology. The findings of the studies reported here complement Lohse’s (2021) discussion of the inertia and conservatism to which he attributes the fact that there has not been more progress on the shift to non-animal studies in biomedicine broadly. In the heightened political context of chemicals regulation, inertia is exacerbated by the stakes involved in the use of scientific evidence. I focus in on the perceptions of participants in the domain of regulatory toxicology, which is responsible for a significant percentage of the animals used in research. As such, the paper is primarily empirical, and aims to set out what are the main challenges of the shift to non-animal methods from the perspective of those involved in the science and regulation of chemicals on a daily basis. Firstly, however, I turn to an outline of the AOP Framework and the approach to toxicity testing that is associated with it. 

## The AOP Framework: an outline of its main features

The AOP (Adverse Outcome Pathway) Framework is a conceptual framework for gathering and organising mechanistic data and information in an adverse outcome pathway. The AOP framework is instantiated most significantly in a social media platform that was initiated on the crowdsourcing model of Wikipedia, that is the AOP Wiki. The felt need for the framework and for the collaborative effort of sharing knowledge about pathways and their component parts is that this knowledge is dispersed across publications, and more progress could be made if researchers pool their data, and their knowledge. The AOP Framework is supported by the OECD, and is gradually becoming more recognised as a useful way of developing mechanistic pathways for researchers; it is still in the process of garnering acceptance by potential users of this knowledge for decision making ( Carusi *et al.*, 2018) In the perceptions of potential users of the AOP Framework, the AOP Framework is often not distinguished from the mechanistic and non-animal approaches that are strongly associated with it (even though AOPs often combine animal and non-animal methods), and this is reflected in the research in both projects, where respondents would answer in the terms of one or the other. Another aspect of the AOP Framework and Wiki is that it is also strongly associated with an interdisciplinary approach, and aims to facilitate and support interdisciplinary collaboration, since a whole pathway (and most often the network in which it is embedded) requires input from specialists in many different methods, and input from several disciplines.

## Methods

The research reported here was conducted over a 7 year engagement with the JRC Unit for Chemicals Safety and Alternative Methods, a unit of the European Commission tasked with progressing non-animal alternatives in chemicals safety testing. The engagement took place firstly through Wellcome funded research to examine the use of social media by scientific communities (2016–2017); it continued through a project commissioned by the unit to investigate the challenges experienced by regulatory toxicologists, risk assessors and risk managers in industry and in governance, in accepting the evidence of non-animal methods.

Research on the first project initially focused on the community’s use of social media; however, in the course of ongoing research with the community, it soon became clear that this could not be disentangled from the encompassing vision for toxicity safety testing. The research included 14 interviews with members of the community, both in Europe and the USA. The interviewees were identified through recommendations from the AOP Wiki core team, and through a perusal of key documents relating to the AOP approach, with the aim of getting representatives from regulators, industry, and academic researchers. We also read through the key documents of the community, attendance of training and other workshops, including the 2017 SETAC (Society for Environmental Toxicology and Chemistry) Pellston Workshop dedicated to the AOP approach, which resulted in the paper Carusi *et al.* (2018). This part of the research is described in section 3. This was the beginning of a more participant action approach, which I have continued in ongoing collaboration with the community. While Carusi *et al.* (2018) includes a stakeholder analysis, ongoing work aimed at a deeper understanding of stakeholders working in risk assessment and risk management in industry and governance settings. This research was conducted through a survey, focus groups, interviews. The study consisted of a survey with 146 responses; 5 focus groups; and 19 interviews (9 industry participants; 9 governance agency participants; 1 NGO. The focus groups were held either at regulatory conferences, or at industry offices gathering risk assessors of particular industries. We had 54 participants in total at the 5 focus groups (See Carusi *et al.*, 2022a, section 2.1.1 for details). In both studies, interviews and focus groups were transcribed and thematically analysed; the themes were partly identified through the topic lists used for interviews, focus groups, and the open questions in surveys; and through an inductive grounded approach: the transcripts from interviews and focus groups were coded through an interpretative process that attempts to stay as as close as possible to the participants’ own words and terms, and not to impose pre-set hypotheses and theories. This methodology was particularly important in this study as the objective was to discover what are the issue of the shift to non-animal methods for the research and risk assessessment stakeholders in chemical toxicity testing. The approach of grounded theory does not assume the neutrality of the interpreter, requiring techniques such as a constant return to the data to cross-check that topics and themes that seem to be emerging are indeed coming from the data; and self-reflection (see for example Corbin & Strauss, 2008).

Before describing the findings of this research, I briefly sketch out the background for the perceived need for a major paradigm shift in chemicals safety testing.

## Results

In the first part of this section, I discuss the results of surveying the literature around toxicity testing, guided in the first instance by the documents (articles, publications, and other) that participants pointed us too, while also contextualising this further in broader debates. In the second part, I discuss the results of the empirical research in interviews, focus groups and surveys.

### A paradigm shift in science for chemicals regulation

Legislation requiring the use of animals for testing chemicals before they are released was first enacted in the US in 1938, following the death of 73 people caused by the drug sulfanilamide –whose active principle had been dissolved in diethylene glycol the chief component of anti-freeze and extremely toxic ( Gaw, 2015). This law was strengthened by an amendment in 1967, requiring testing not only for safety but for efficacy. Swaters *et al.* (2022) analyse why these public health disasters gave rise to the view that substances should be tested on animals. As the use of animals for biological, physiological and medical research had become established in the laboratory approaches of the 19 ^th^ century ( Franco, 2013), it is not surprising that animals became entrenched in toxicological research and governance too. The numbers of animals used for chemical safety testing of drugs and products is huge: between 1500 and 3000 animals will be used to bring a pharmaceutical compound to market ( Nuffield Council, 2005), and an estimated 10 000 animals will be used for a pesticide, against a background of an estimated 100 million animals being used each year for research and testing ( Taylor *et al.*, 2008). The numbers in the EU show that 18% of the 9 million animals used in 2018 were used in toxicity studies ( Swaters *et al.*, 2022). The 2010 EU Directive already cited shows a commitment to see significant downturns in the numbers of animals used, reflecting ethical concerns. Ethical arguments against the use of animals in research and testing are gaining ground through the efforts of animal welfare organisations and animal activist groups, and public opinion is slowly edging against the use of animals; the 3Rs of animal research (refine, reduce, replace) are frequently invoked as an aim of various biosciences initiatives ( Carusi, 2016).

The ethical-legal urgencies by themselves are not the main drivers of the need perceived by many in regulatory toxicology for a major shift away from established methods. The arguments for shifting away from animal experiments for chemical safety testing that seem to carry more weight among reseachers in this field are a combination of epistemological and pragmatic considerations ( Hartung, 2009). First, the sheer numbers of industrially produced chemicals would take too long and be too costly to address only through animal tests ( Villeneuve & Garcia-Reyero, 2011); second, the limitations of extrapolations from animals to humans, and from any one species of animals to other species of animals, as the variability of biological responses across species becomes increasingly obvious ( Dutt & Latham, 2013); thirdly, the kinds of chemical substances we are now dealing with – such as endocrine disruptors – and the complex combinations in which they exist in real world environments, mean that traditional ways of thinking about the safety or risk posed by chemicals are no longer adequate to the way in which these chemicals interact with bodies and environments ( Bradbury *et al.*, 2004; Vogel, 2008; Vogel, 2013). Fourth, the claim is made that observational studies carried out using animals blackbox complexity rather than analysing it, which is the aim of molecular, mechanistic approaches. Lastly, the claim that opening up the blackbox of animal-based observational studies to a molecular mechanistic view will be more efficient, both in terms of time, and of expense, and will be better able to cope with real-world complexity, characterised by multiple chemical exposures, complex layers of chemical and biological interactions, and low doses and long-term exposures ( Garcia-Reyero & Perkins (2011); Villeneuve & Garcia-Reyero, 2011).

Toxicologists working in public oversight and regulation of chemicals are keenly aware of the need for an overhaul of the whole approach to chemical safety testing ( Sanderson & Solomon, 2009). The response by government bodies to this challenge is exemplified in the US Environmental Protection Agency’s request for an in depth report on how new tools, methods and approaches in biological sciences and biotechnologies, could be mobilised to address some of the perceived deficits of traditional methods for testing the safety of chemicals. In 2007, the Committee for Toxicity Testing and Environmental Agents produced the requested report addressed to the relevant US governacne advisory bodies: the National Academies of Sciences, Engineering and Medicine. This report ‘Toxicity in the 21 ^st^ Century: A Vision and Strategy’ heralds a ‘paradigm shift’ in chemical safety testing ( Krewski *et al.*, 2010), a term as often invoked as contested in scientific and technological innovation. It is worth noting that here its use stresses the urgent need for changing how chemical safety testing is carried out at the science/society interface where knowledge about chemicals meets the regulation of chemicals. The pivotal methodological upheaval is the shift from testing chemicals on animals, to testing them through a number of biotechnologies that have emerged in the eight decades since animals started to be used, in particular a shift towards human cell lines (more easily available thanks to stem cell technologies); high throughput *in vitro* methods; the technologies for ‘omics’ research: genomics, proteomics, metabolomics, etc.; and the use of supercomputing for computational modelling and simulation. In this publication, the claim is that the overarching theoretical and methodological framework needed is similar to systems biology, in its reliance on molecular methods, and the array of instruments and quantities of data that this implies. In this respect, it shares many of the characteristics of toxicogenetics and the moleculerisation of toxicology, as described by Shostak (2013).

### Study 1: the development and use of Adverse Outcome Pathways

The first study we undertook focused on the AOP approach, and its user community. As noted above, the AOP approach is closely associated with mechanistic methods in general, as well as with the promotion of non-animal methods, and thus it can sometimes stand proxy for these in discussions. It can garner both support and oppobrium depending on pespectives and interests. On one hand, it is supported by animal welfare NGOs as a powerful tool for achieving the 3Rs of the use of animal for chemical safety testing. For example, funding institutions such as the UK National Centre for the 3Rs (NC3Rs) have channelled funding towards the development of AOPs ^2^. On the other hand, the AOP approach has been accused of serving the purposes of industry, even of having been hijacked by industry ( PAN, 2016). It is well known that those with commercial interests have developed several techniques to bend science and to exploit uncertainties in their interest ( Henry *et al.*, 2021; Krimsky, 2004; Mcgarity & Wagner, 2008; Michaels, 2008; Oreskes & Conway, 2010). Quality controls on the AOP Wiki include indications of the level of approval status of uploaded AOPS (up to OECD endorsement), peer review, disclosure of the identity of peer reviewers, and of their comments. In this way, efforts to ensure that the provenance of evidence and of potential disagreement around it, are transparent. This is an area of ongoing development of the AOP Wiki. However, some claim that the coexistence of AOPs and traditional apical animal testing can increase the scope of uncertainty around a chemical, an uncertainty behind which industry is well known to hide and to introduce enough obfuscation to undermine claims of potential harms. Some of the methods harnessed by AOPs, such as PBPK models, are accused precisely of this ( Brown & Grossman, 2015). A critical mass of information about chemicals shared on the knowledge base may fill in data gaps, but it may also be perceived as increasing the uncertainty around chemicals that are otherwise thought to be well understood, as it has been accused of in a report by the Pesticide Action Network. ( PAN, 2016). Shostak (2013) discusses similar concerns in relation to toxicogenetics in chemicals regulation.

Unsurprisingly, there is a great deal of critical questioning of the approach by stakeholders speaking for broad public interests. For example, the central criticism of the AOP approach in the PAN report is that it has already been misused by corporations that use mechanistic information to undermine established (animal) studies that show adverse effects. For example, the authors of the PAN report claim that

A massive misuse of AOP can be foreseen if a chemical company fights to get their chemical on the market, no matter how. Currently the first examples of this misuse can be observed already in the initiative of the fragrance industry to predict adverse effects solely based on assumed similar chemicals of known toxicity.
  Also in the EU approval of pesticides the first examples can be observed; Health DG SANTE [the European institution responsible for pesticide testing] even allows overruling of adverse outcomes observed in animal testing. ( PAN, 2016: 3)

However, these criticisms are not lost on the proponents of the AOP approach. For example, one respondent in our study – a government expert developing AOPs – stated:

Quote 6
“When I started here at the XXXXXX I was in charge of running a hazard assessment programme where we actually were developing hazard assessment for *in vitro* chemicals and the most difficult discussions that we had was in animal test results what was the statistical significance of animal test results. If you saw effects but they were not statistically significant, immediately toxicologists become unhappy, they become uncertain, they have trouble discarding effects seen in an animal just because of statistical considerations. It makes them uneasy. […]Yes, you observe something and you discard it. That's counter nature [emphatic]” [R7]

The perceptions of animal studies therefore pull in opposite directions: on the one hand, they ought to be cut back for both epistemic and ethical reasons; on the other, they are the current ‘guarantee’ of a certain standard of chemical safety testing that is still seen as a restraint on industries to use potentially harmful chemicals. Clearly, there is a full-scale methodology war going on in chemicals regulation, made all the more intense in this context where regulatory decisions are in the balance, and there are huge economic, environmental and health stakes.

Several of the interviews with researchers in academia and in other governance and state institutions, people in regulatory bodies and in industry, showed that even among this group that could be considered ‘converted’ to the AOP approach, and broadly in favour of sharing knowledge via the AOP Knowledge Base, there are disagreements and tensions over methodologies. The more serious of these have to do with linearity and complexity: many interviewees complained about a perceived linearity of the approach, in the way that pathways are represented (as a sequence of boxes and arrows, representing key events, and relationships between them). This complaint sometimes tracked disciplinary differences, with those in biological sciences more likely to say that the approach, and the AOP structure, is overly linear and unable to cope with the complexity of biological processes, but also a useful simplification; it also sometimes tracked differences across sectors, with some users from industry making a similar complaint. This has continued to be a live debate among stakeholders in AOPs. Complexity and linearity frequently came up in discussions and conversations at workshops we attended; that chemical safety testing has to be capable of dealing with complexity is agreed on by all; rather the disagreement is about where and how the complexity is best represented, in the whole organism or in networks of molecular pathways. For proponents of the former, whole organisms *are* complex, and therefore the outcomes of results using them as experimental models do take complexity into account. Proponents of mechanistic approaches claim instead that the complexity of the processes is blackboxed in whole organisms, and while the results of tests are brought about through biological complexity, there is not an understanding of the mechanisms whereby the result came about. [Personal communication]. The term ‘linear’ as used by different people in the community (as evidenced in interviews, and in workshops and meetings) is somewhat ambiguous, and it can be used as a criticism (an inaccurate representation of complex biological complexity), or the opposite, as simplifed and thereby able to show the most important information from a regulatory perspective. A further layer of ambiguity is that the visualisation of AOPs is sometimes understood as a knowledge management tool, and sometimees as a representation of a biological pathway, and it garners more criticisms when it is seen as the latter. Disambiguating between these understandings is task of ongoing development. Disentangling simplicty and complexity is at the heart of ongoing development of the approach, with one out of the four workgroups of the recent Pellston Workshop on the topic of the AOP approach dedicated precisely to how to develop AOPs as networks ^3^. There is, however, a tension between the need to represent AOPs as networks on the Knowledge Base, and its purpose to act as a bridge between science and decision-making, including for regulatory purposes, because the greater the complexity, the more difficult it is to synthesise the knowledge about a chemical (or chemical interactions) in a way that is digestible and useable for regulators. A member of the community (a scientist in a governance institution) described the quandary as follows:

Quote 7
“When it comes to the endocrine system […], there is not one pathway, [but] many pathways that interact with each other, and this is actually the AOP unit, and [it] is actually quite complicated and very difficult to put it in a small sequence of events. On the other hand though, you don't want to make it overcomplicated for the regulator, who would like to see a clear sequence of events, that would help them make a decision. I don't expect all the regulators to have a very deep knowledge of the endocrine system of different fish species ... of all the animals we're trying to protect. So it's a necessary evil in so many ways, the linearity, and what we suggested as a group, was that we should have different levels of organisation for different purposes. So with the wiki we should have a simple one, for lay people who don't need to understand all the complications, but by clicking on a relationship, can go one level deeper and see more of the complexity, see the other pathways there cross talking, ... and for those who are really into hard core science can go even deeper and see the hairy monster. ... so it's a necessary evil, it's not necessarily correct but it's fit for purpose.” [R13]

Here too, the simplicity/complexity of representation and of the process are very closely interconnected, but can cut across each other: the linearity (in the sense of simplification) of the visualisation should not be taken to imply linearity (in the sense of uni-directional causation).

Members of the AOP community tend to come from different disciplinary backgrounds in different sectors. As such they have diverse views about evidence, proof and validation. Sharing a knowledge base, with all the interactions and meetings this implies, provides an opportunity for discussion and dialogue which brings to light basic orientations and assumptions about evidence. For example the following two quotes from two different respondents show up some of the tensions around what it might mean to ‘prove’ a claim:

Quote 8
“When I say prove, in the situation when you have sufficient knowledge of the AOP you know if a chemical interferes with this cell line, then the next thing is going to happen at the organ level and the next thing is going to happen at the animal level, and you have the test results to prove all that, you have in vitro test results, you have intermediate test results, animal test results.” [R7]
Quote 9
“… so we observe processes in these cells, and we deduce that this correlates with ongoing in vivo, but we don’t really have the proof, we don’t really understand what does it mean what we observe.” [R6]

This is also seen as a useful process that enables people to come to different understandings, as indicated in the quote below.

Quote 10
“Interviewer: Among the different disciplines that you've interacted with have you ever had experience of people disagreeing that the AOP captures the most important concepts.
Respondent: Yes. All the time [laughs]. This is the joy of it. So each community will have a different view on how much information you need in order to capture the essence of the process, and in mathematically modelling those different hypotheses, you get closer to understanding what information is useful and what's not. But of course everyone comes with a completely different perspective on what's important because they start from what they know. But this is the joy of it, because by having those discussions I've seen people change their minds, I've seen people reviewing evidence outside their field or at least consider evidence in a different light that ultimately moves people forward and focuses them more on where there's consensus rather on areas where there's disagreement, it moves people to consensus quicker.” [R5]

In summary, there clearly is controversy around methods and evidence when a new approach such as the AOP approach is developed and proposed to stakeholders in chemicals regulation. This controvery is fueled by mistrust of the ways in which interests may underlie the choice of methods, but not only: those who are not a priori hostile, and who work to promote alternative methodologies are not a homogenous group of people all sharing the same views. This chimes with recent research showing the complexity of interests in framing evidence concerning chemicals ( Thomas, 2021). Seen as a knowledge management tool, the AOP framework serves both scientific and social purposes. As a scientific tool, it is a conceptual framwork for thinking about toxicity on a molecular level; as a social tool it gathers, organises and shares data, evidence and knowledge. This means it also shapes the space of social interactions around the uploaded pathways: affording collaboration, but also negotiating ways in which to achieve transparency, an ongoing work in progress. Potentially it plays a powerful role in mediating discussions and research across methods and approaches ( Carusi *et al.*, 2023. This may bolster it in its bid to be an ‘honest broker’ in the controversies and tensions between diffeerent stakeholders. They potentially play the kind of consensus building role that Shostak discusses in relation to shifting towards a molegular regulatory science (2013:160). This is discussed in more detail in the next section.

### Study 2: AOPs in the broader context of chemicals regulation

In this section I discuss findings of a study commissioned by the JRC Unit for Chemicals Safety and Alternative Methods. Carusi *et al.* (2018) points to 4 main stakeholder groups that are crucial to the AOP approach. While academic or academic-related researchers have been dominant contributors in the AOP community, it was felt that there was still a lack of understanding of stakeholders in regulatory agencies and in industry. The study targeted risk assessors and risk managers in these settings, and in the design of the questions and the channels through which it was disseminated, although the academic and NGO sectors were retained in questions eliciting information about sectors in the survey. From the outset of the study it was difficult to find out where risk assessors and risk managers were likely to be employed, and also difficult to get quantitative information about the size or distribution of the pool, and we therefore left it as wide as possible. It is interesting to note how little is known about the chemicals sector broadly. Risk assessors and managers work in a wide variety of contexts; they also very different roles within their employment setting, with varying levels of power and influence, and with varying levels of scientific training or involvement in toxicological research. This variability seems to be a feature across industry and regulatory agencies, and a better understanding of the make-up of these stakeholder groups would be very valuable. The study was also limited by the geographic distribution of our respondents, who were mainly concentrated in Europe and the US. ( Carusi *et al.*, 2022a, Part One]. One salient point of the information that is available is that there seems to be a generational change occurring in those employed in these roles and positions ( Carusi *et al.*, 2022a, section 1.3). This change may be the single factor most likely to bring about a substantial change in the science and practice of chemicals regulation.

We chose not to focus narrowly on the AOP framework, but more broadly on the challenges of regulatory toxicology, and non-animal methods, looking at AOPs as a subset of these. The survey contained many free text boxes for commentary, besides the quantitative responses to questions. We also carried out focus groups at conferences or through associations known to have a high number of participants from industry or governance, as well as one-on-one interviews. (See Carusi *et al.*, 2022a, section 2.1.1).

The main challenges facing chemicals regulation, according to study participants, related to data, the state of the science behind regulation, and interactions between the sectors. The absence of data for an increasing number of chemicals circulating in environments through industrial products is a major concern, as is the fact that when there is data, it is dispersed and non-standardised. The state of the science was also a focal point for a majority of participants, that is, aspects relating to forms of evidence, and methodology in general. There is a lack of agreement over which methods produce the most reliable forms of evidence, or which show the most promise and are worth pursuing. This lack of agreement was rife across both regulatory and industry settings. Participants in both sectors pointed towards the absence of clear and up-to-date guidelines for toxicity testing; differences in the way in which data are interpreted by different bodies without transparency regarding why those differences arise. A very common theme that emerged from respondents in Governance roles and in Industry is that the science that is used for regulatory decision making regarding chemicals lags behind new advances in scientific methods and approaches. Below are two (of many) examples of comments made on this point by respondents working in governance agencies or institutions:

“Despite the fact that science is running … we are doing risk assessment basically like we did it 30 years ago.”
“The way we do safety assessment today is the way we’ve done it for decades. We’re in a world of technology and science that has rapidly progressed to the point where lots of the cars, phones, etc, are very different than they were 50 years ago. And our science is not as advanced as it ought to be. All of our investment in technology and science ought to enable us to do this in a way that looks very different from the way we’ve always done it and we’re not there.”

Another concern frequently expressed by participants in open commentary is that regulation is overly political and insufficiently informed by science, with many pointing to a bigger role for academia as a possible measure to address this.

Non-animal methods clearly evoke strong feelings among all study participants. All sectors – including regulators in governance settings -- felt that the fact that non-animal methods are not required, nor accepted by regulators is the main challenge facing non-animal methods. Attitudes towards these are inter-related with perceptions of the main challenges facing chemicals regulation in general; in particular, those who believe that current regulation is out of step with current science tended also to believe that there is a bigger role for non-animal methods in chemicals regulation than is accorded them. There are two main underlying factors: the first has to do with questions relating to the methods and their usefulness in toxicological research; and the second has to do with the regulatory paradigm, that is, the dominant ways of conceptualising evidence standards, and for setting the criteria that data and evidence should meet. For many decades, the dominant regulatory paradigm for data and evidence are set by animal studies, and other kinds of studies are still expected to fit into that paradigm. This underlies many of the challenges relating to methods faced by non-animal studies. The link between these two main factors is validation. The clarion call is for *validated* non-animal studies or alternative methods; however there are not yet widely agreed validation standards. This is a widespread issue across the board for toxicity tests, but it is particularly important for non-animal tests. In recognition of the need to validate non-animal studies, the European Union Reference Laboratory for Alternatives to Animal Testing (EURL ECVAM) ^4^ was established in 2011, as required after being directly called for by the EU Directive on the Protection of Animals Used for Scientific Purposes (Directive 2010/63/EU). ^5^ A central activity of this centre is to validate non-animal alternatives, and a detailed validation submission process is described, with a strong emphasis on peer review and the ability to comment in a transparent way by all stakeholders. Thus the process is framed as an ongoing conversation before a recommendation (rather than a final decision) is made. The actual criteria for validation tests are however still being discussed, and still not standardised or universally accepted; an overview of six broad validation frameworks is given in Patterson, Whelan and Worth (2021), before they present their own based on credibility rather than validation (a word which is highly controversial in and of itself). There is no dearth of efforts to gain agreement on validation (or whichever other term is used as a proxy for acceptance); however there is still an absence of widespread agreement among the very different groups who make up the stakeholders. Currently, integrating different methods and approaches is gaining traction as an approach to validation, most prominently in the Integrated Approaches to Testing and Assessment (IATA). In any event, IATA or the integrated approach, will definitively shift away from animal studies as the ‘gold standard’ for validation. Increasingly there is an acceptance that comparison with animal studies should not be the ultimate criterion for non-animal alternatives, since no single non-animal study can ‘mimic’ animal studies. A one-to-one comparison is not the most fruitful target to pursue in alternative methods; however integrating many different studies can give a broader picture of toxicity, and may gradually diminish the role of animal studies, or in some cases push them out, as has occurred for skin sensitisation ( Strickland *et al.*, 2016, see also Cronin, 2017).

Participants in the study were almost unanimous in seeing non-animal tests as being too far from validation. This is also echoed in other discussions, for example as reported by Cronin (2017). Even those most well disposed towards non-animal or alternative methods still spoke of them as needing to be developed for toxicological purposes, and especially for regulatory toxicological purposes. That is, everyone seemed to agree that except in a few cases, these methods are somewhere on a spectrum of meeting their potential of usefulness, rather than currently being ready for full regulatory deployment. Several participants pointed to a lack of investment in non-animal alternatives as a major obstacle to them meeting their full potential. The lack of investment in turn was mostly attributed to the regulatory context: for example, the ‘inherent conservativeness’ of regulators, the lack of training in new methods, or the pressures that regulators in turn are under from policy makers block investment into developing these methods. In short, many of the factors already discussed in the previous section.

When a particular study is excluded from the repertoire of studies required or accepted by regulators, they tend not to be done at all, rather than done and not included in the submission dossier to regulators. For example, one research participant actively wanted to pursue a non-animal alternative for a chemical they were researching, but could not justify the costs of doing this to the client, who would only pay for research leading to data required by regulators.

Only some industry participants are able to invest the R&D funds into these methods, and often this in turn results in issues over data proprietorship (and therefore lack of transparency). On similar lines, comments were frequently made about the non-standardised nature of non-animal studies and the data deriving from them; however, standardisation is dependent on regulator acceptance and input. Other comments referred to the *relevance* of non-animal studies, where relevance is a question of whether they succeed in answering questions set by the regulatory requirements — for example, in terms of exposures, dose-response, etc. Very importantly, many participants pointed to the need for non-animal studies to be *integrated* with animal studies, as integration is a way of showing relevance. Some others were more pessimistic about the potential of non-animal studies in view of the entrenchment of animal studies. For example:

“But even if you develop this parallel AOP in tested animals or in humans by extrapolation or modelling is that the tendency goes to stay at the animal level. So the knowledge we try to gather to fill the gaps, to make sense in a risk assessment, sometimes it’s actually useless because the focus is on what happens in the animals.”

Several participants pointed out that non-animal methods often lead to an increase in animal studies, which is counterproductive if the main motivation for the use of non-animal methods are the 3Rs of research (reduce, refine, replace). This occurs because the results of non-animal studies – either negative or positive concerning the toxicity of particular substances – leads to a demand for tests to be repeated, using animal studies.

“If you use [in vitro studies for example], it as a screening test — consequence is that you might catch a number of positive reactions and conclude that there is something going on. And regulators will want full battery of existing tests, because of precautionary principle. So more animal studies will be needed.”
“Regulators tend to be very conservative. Screening will catch potential unwanted biological activity from a chemical or something else, but what if this is only a part of the information? Regulators will say, well you never know, and industry will be faced with massive further tests.” [IND]

There are genuine scientific questions behind the interpretation of data from non-animal studies, as pointed out by the USEPA joint response to the survey question on the main challenges of non-animal methods:

“Interpretation of results: use within risk assessments and subsequent regulation of chemicals; coordination and consistency of in vitro testing.
  Many methods/models have limited applicability domains. More work is needed on: integrating information into risk assessments and translating to equivalent *in vivo* doses; consideration of toxicokinetics is difficult for many reasons in *in vitro* studies; *in vitro* results can give false negatives and false positives; characterization of uncertainty for non-animal methods.“

Other research participants echoed the points made previously about regulation being perhaps ‘behind the science’ on the acceptance of non-animal studies:

“Non-animal approaches have been shown to give more relevant and mechanism based information on the chemical or mixture, however there is significant lag in the regulatory acceptance of these methods” [ACAD]

However, another participant, also from Academia, pointed out the difficulties of extrapolating from non-animal studies – here, in vitro cell-based assays – to the whole organism:

“There is very little appreciation for the complexity of cell-based assays and the difficult in extrapolating from one cell type to another, much less to a whole animal including human.” [ACAD]

Such lack of consensus on the scientific standing of non-animal studies was pervasive. There is scientific uncertainty associated with these tests, but also different levels of toleration of uncertainty in the different sectors.

“New methodologies that are often pushed by academia and institutes that are keen to push new techniques. Eg JRC. Being interesting is one thing, but being useful in a regulatory context is to push for more and more testing, which is not the idea, So you have the opposite effect. Because don’t accept uncertainty at the same level at academia.” [IND]

Several participants across all sectors pointed out that there is also uncertainty in the case of animal studies, and that the gold standard of animal tests is not as ‘gold’ as it is assumed to be. For example, a participant at a leading governance agency stated this about animal models:

“On the animal studies, I would say [….] they are a model. The data only describes the model. Reality is different. The big problem of the animal model is that it is the less validated — how to validate it? [Its results] would work for some substance, but one that can be metabolised for the rat and not for the human … [this is] very difficult to validate, and actually we don’t have a formal validation as for example we now require for in vitro. Also animal models are ethically and resource demanding. They cost a lot. How can you screen 5000 chemicals using the standard pesticide programme for example?”

This participant raises the point that in fact more demands for validation are made from in vitro studies than are made for animal studies. However, the very familiarity of animal tests makes the uncertainty more tolerable — possibly they are a case of being the ‘known unknowns’, whereas the uncertainty of non-animal studies are ‘unknown unknowns’.

Yet other participants were convinced that there the shift towards mechanistic methods could potentially bring about a shift in the regulatory paradigm itself, one that does not regulate on the basis of risk but on the basis of hazard. This quote is from a participant in a governance agency:

“But if you go to the IATA [Integrated Approaches to Testing and Assessment], then I see a role for everything. . A role for mathematical model. I see a role ... but in the context of all the data. This to me is the mechanistic drift. Understanding the mechanistic drift is to me the key role of the AOP. Then if the mechanistic drift is changing the regulatory mentality from hazard to risk factor, well then you change the data requirement, because you are not looking for hazard, you are looking for risk factor. The key event is the risk factor, the adverse outcome is the hazard. But if you want to substitute the hazard with something different, it will take a lot of time.”

This shift would indeed be momentous, as a shift towards hazard rather than risk tends on the whole to be more precautionary, and to exclude more chemicals from approval ( Lofstedt, 2011); and thus arguably would be counter to industry interests.

## Discussion

Disputes and disagreements in chemicals regulation, like other fields where policy and governance play a crucial role in protecting populations against risk, can occur for many different reasons. Bozzini (2020) shows how one of the most important controversies in the field of recent time, around the carcinogenicity of glyphosate, has resulted from the different norms used by the different bodies coming to judgements on the topic. These norms are institutional in their nature, and are frequently set out in guidelines and other material. There is also another level that shapes the epistemic space of a field, including the general attitudes towards what counts as reasons and evidence. These are often not completely formalised although they exist within, across, and overlapping with institutions. This is what this paper has focused on, in its focus on what people ‘on the ground’ believe about the prospects of shifting towards non-animal methods. The two studies reported here are modest in their scope; they are relatively small studies, focusing primarily on hearing the voices of a limited number of people working in the domain of chemicals regulation in various capacities. It is difficult to draw generalisations. Some points that are deserving of more attention in ongoing research are the following:

- Participants seemed to be in broad agreement that chemicals regulation is in need of fundamental changes in the approaches to toxicity studies and toxicology in general that regulatory decisions and policies are based upon. This was attributed to the number, combinations, and pervasiveness of industrial chemicals in products and in the environment. Molecular, mechanistic approaches were acknowledged to be at the heart of this shift; these also often are non-animal approaches (but not exclusively). The legislative requirement in Europe to phase out the use of animals also plays its part in increasing the use of non-animal methods, but it seems to be secondary to these other demands.
- The methodology wars that can occur in scientific domains are exacerbated by tensions between interest groups, and widespread mistrust between sectors. This was apparent particularly in the interviews and focus groups of the second study which included ris assessors across regulatory and industry contexts. The mistrust is further fuelled by the many uncertainties involved in the shift towards mechanistic approaches. For many research participants, a major reason for these uncertainties is that they remain relatively underdeveloped with respect to how they would be validated.
- The shift in methods and approaches requires a concomitant shift in the framework for validation, or the criteria of acceptance of evidence for regulatory purposes. Key voices that we heard agreed that the age of the ‘gold standard’ of animal tests seems to have passed, but it seems not to have been definitively replaced. There are many different initiatives in this regard, such as Integrated Approaches to Testing and Assessment promoted by the OECD, and in which the AOP Framework initiative also participates. Even though the OECD is a major international player in forming policies with regard to chemicals, there is also a great deal of geographical variation in the degree to which initiatives are shared internationally. In addition, the OECD has its own permeability to influence by economic and political interests ( Lanier-Christensen, 2021), which are particularly crucial to consider as this large scale shift in validation guidelines and standards is occurring.
- There seems to be a very big gap between the level at which these initiatives are carried out, and the realities of those people on the ground, who are carrying out risk assessments and doing risk management, or providing data for these. This may be due to a training gap; or even a generational gap ( Carusi *et al.*, 2022a, section 1.3). A hypothesis worth pursuing is that the acceptance of new approaches, including non-animal methods, may be ushered in through generational change as those recently and currently going through training in regulatory toxicology will be exposed to methods now considered alternatives, and also have or be exposed to a shifting set of values.
- The research across different groups showed that there is not a big difference in attitudes to non-animal studies between those working in industry and those working in regulatory contexts. This can be attributed to a number of different factors, including that there is a greater complexity in the makeup and interests of stakeholder groups than the industry/governance binary gives to believe ( Thomas, 2021). Perhaps most importantly there seems to be widespread agreement that the science on which chemicals regulation is actually conducted has not kept up with new developments in biosciences. Once again, a generational shift may see this lag close up.

Finally, two points that are made from my own perspective as participant observer in this domain, and as a theorist of social aspects of chemicals regulation, regarding the lack of trust in this domain, and the pivotal role of validation.

The lack of trust between sectors (despite the previous point) is a major stumbling block in chemicals regulation. There are many obvious reasons for this, particularly in view of the health, environmental and economic interests at stake. However, particularly unfruitful disagreements around the different approaches and methods occur when there is an absence of any shared way of talking about the criteria for the relevance, robustness, reliability and acceptability of toxicity and toxicological studies. It is very difficult to compare conflicting decisions made about substances, because they are often based on very different understandings of evidence, of toxicity, and of hazard and risk (the controversy around glyphosate being a case in point, see Robinson *et al.*, 2020). Establishing a framework for shared understandings is the topic of the Science for Policy report issuing from this study ( Carusi *et al.*, 2022b). This does not mean consensus all the way to a final judgement; however it does require at least transparency and understandability of the evidence that is provided for validation of the methods used in the essential steps leading towards judgements. This is where something like the AOP framework may come into its own, since it sets out precisely a framework, for articulating knowledge around toxicity testing in such a way that it not only is findable and accessible, but is also understandable across disciplinary and other contextual divides.

Regarding validation: While *validation* sounds like it ought to be a purely scientific or even technical issue, which will make explicit (and therefore transparent) what counts as good data and evidence on which to ground a decision, it is finally an issue of what is deemed acceptable, reliable, or credible *by stakeholder communities*. Validation thereofore always has a social aspect ( Carusi, 2014; Winter & Carusi, 2022). This is not to say that there is not a scientific fact of the matter, but rather that what this fact turns out to be depends on which is the question that must be answered. The very fact that current scientific tests focus on *exposures*, for example, means that some methods will be more likely to be able to meet the requirements of validation than others, with exposures being mapped onto dose response curves, which are achievable and observable in test conditions, and also highly constructed ( Demortain, 2019). Dose response curves are an artifice of a paradigm of toxicological testing based on animal studies; moreover, setting the question in terms of exposures, is itself not a purely scientific decision, but is one that is already informed by social, economic and political demands. There has already been a prior choice to tolerate chemicals in products, homes, workplaces, and in the environment, and to frame toleration in terms of exposure and permissible exposure levels ( Boudia and Jas (2013) which inevitably different jurisdictions and different countries will construe differently). In my view, a more explicit and deliberate attention to the social role of validation, both among the stakeholder communities and the broader context of what it is for, will greatly help to push this conference forward.

## Conclusion

The sciences that inform chemicals regulation are undergoing a major shift towards a more mechanistic approach that is less reliant on animal studies. This is in line with the molecularisation of biosciences; and also in line with different pressures to reduce animal testing drastically, for ethical, legal, pragmatic, and epistemological reasons. This shift is beset by many uncertainties with their concomitant risks. Frequently anxieties around this are expressed as a demand for validation. The significant reduction of animal testing depends on a re-formulation and re-constitution of what counts as validation in chemicals toxicity testing; if it is to have any hope of garnering the trust of major stakeholders, active interventions to bring about shared understandings of the forms of evidence that can support validation are also needed. Chemicals regulation is intensely political, so it is not surprising that validation, that sits on the knife-edge between science and society, should require a form of social engagement and organisation to ensure not just that it is done, but that it is accepted by key stakeholders.

## Ethics statement

The research for Section Two of this paper was approved by the University of Sheffield Institutional Review Board, on 14/06/2016, no 009282.

The research for Section Three was conducted under the auspices of the the JRC Unit for Systems Toxicology, which does not have an institutional review board. However, all participants were sent an information sheet and consent form, modelled on standard university institutional ethics board requirements. I can supply copies if needed.

## Data Availability

Contact a.carusi@inter-changeresearch.com for access; data will be shared as per agreement with participants. Figshare: Non-animal methods for toxicity studies and chemicals regulation, https://doi.org/10.6084/m9.figshare.23898948.v1 ( Carusi, 2023). This project contains the following extended data: Crowdsourcing for Health Topic List.docx Theme lists.docx Codes.docx AOP framework study, questions for interviews and focus groups.docx AOP-Survey-Questions.docx Data are available under the terms of the Creative Commons Attribution 4.0 International license (CC-BY 4.0).
