# Supplementary material for: Chemicals regulation and non-animal methods: displacing the gold standard
Source: Wellcome Open Res. 2024 Sep 2;9:167. Originally published 2024 Mar 20. [Version 2] doi: 10.12688/wellcomeopenres.20581.2 (PMC11391182; doi:10.12688/wellcomeopenres.20581.2)
Supplement: Peer review history

# Reviewer report: Reviewer response for version 2

**DOI:** 10.21956/wellcomeopenres.25305.r96746

**Published:** 2024-09-11

**Authors:** Laura Aliisa Saarimäki

**Affiliations:** 1 Tampere University, Tampere, Finland

The changes applied to the discussion provide the needed clarity.

 I would note that the last paragraph of the section "Study 1: the development and use of Adverse Outcome Pathways" still contain the typos I have highlighted in my previous comment, but this is a purely cosmetic consideration.

Is the work clearly and accurately presented and does it cite the current literature?

Yes

If applicable, is the statistical analysis and its interpretation appropriate?

Not applicable

Are all the source data underlying the results available to ensure full reproducibility?

Partly

Is the study design appropriate and is the work technically sound?

Yes

Are the conclusions drawn adequately supported by the results?

Partly

Are sufficient details of methods and analysis provided to allow replication by others?

Partly

Reviewer Expertise:

Toxicogenomics, mechanistic toxicology

I confirm that I have read this submission and believe that I have an appropriate level of expertise to confirm that it is of an acceptable scientific standard.

# Reviewer report: Reviewer response for version 2

**DOI:** 10.21956/wellcomeopenres.25305.r96745

**Published:** 2024-09-11

**Authors:** Simon Lohse

**Affiliations:** 1 Radboud University,, Nijmegen, The Netherlands

The revision of the article addresses all major criticisms I had. In particular, the revision of the methods and discussion sections is very well done.

Is the work clearly and accurately presented and does it cite the current literature?

Yes

If applicable, is the statistical analysis and its interpretation appropriate?

Not applicable

Are all the source data underlying the results available to ensure full reproducibility?

No source data required

Is the study design appropriate and is the work technically sound?

Yes

Are the conclusions drawn adequately supported by the results?

Partly

Are sufficient details of methods and analysis provided to allow replication by others?

Partly

Reviewer Expertise:

Philosophy and social studies of science

I confirm that I have read this submission and believe that I have an appropriate level of expertise to confirm that it is of an acceptable scientific standard.

# Reviewer report: Reviewer response for version 1

**DOI:** 10.21956/wellcomeopenres.22781.r83307

**Published:** 2024-06-12

**Authors:** Laura Aliisa Saarimäki

**Affiliations:** 1 Tampere University, Tampere, Finland

This article by Carusi discusses the perceptions of people from various stakeholder groups on the challenges in shifting to non-animal testing methods in chemical safety. It presents part of the results of research carried out over a seven-year period in the context of two separate studies and their outcomes are presented as a cohesive outlook into the thoughts and perceptions of the participants. The article focuses largely on the AOP framework as the backbone of mechanistic approaches and as the anchor of non-animal methods. Moreover, the concept of validation is raised as a key topic.

 Considering the nature of the study, the results cannot be evaluated for validity or accuracy; they give insight into the perceptions and challenges pervading the field. Many of the topics raised by the participants and further condensed by the author are recognised in individual discussions or specific instances, but rarely brought together nor contextualised as a whole. Hence, although small in sample size, I believe this to be a valuable look into to the field. Understanding these challenges and gaining a broad view on them and their implications is important for advancing science and refining regulations. However, I think it would be valuable to expand the discussion section to indicate how the answers from the interviews/questionnaires link to the points condensed in the discussion. This is something that is lacking in the methodology. How did the author interpret the open text results and decide which parts to include in the manuscript?

 Overall, the article serves in purpose to make the voices of the people involved in the science and regulation heard. It raises topics that need to be considered as the field moves forward, also clarifying why it should move forward.

 Minor considerations:

 The text should be proof-read for one more time. Several typos were spotted, e.g., in the paragraph just before the section on Study 2 in the results (framwork, diffeerent, molegular…) and perhaps some missing words e.g., towards the end of the page 7: “The main challenges facing chemicals regulation, according to study participants, related to data, the state of the science behind regulation, and interactions between the sectors.”

Is the work clearly and accurately presented and does it cite the current literature?

Yes

If applicable, is the statistical analysis and its interpretation appropriate?

Not applicable

Are all the source data underlying the results available to ensure full reproducibility?

Partly

Is the study design appropriate and is the work technically sound?

Yes

Are the conclusions drawn adequately supported by the results?

Partly

Are sufficient details of methods and analysis provided to allow replication by others?

Partly

Reviewer Expertise:

Toxicogenomics, mechanistic toxicology

I confirm that I have read this submission and believe that I have an appropriate level of expertise to confirm that it is of an acceptable scientific standard, however I have significant reservations, as outlined above.

# Reviewer report: Reviewer response for version 1

**DOI:** 10.21956/wellcomeopenres.22781.r83313

**Published:** 2024-06-05

**Authors:** Simon Lohse

**Affiliations:** 1 Radboud University,, Nijmegen, The Netherlands

The article addresses the challenges of replacing animal testing with non-animal methods in the context of chemicals regulation and presents the results of two empirical studies analysing stakeholders' views on these challenges. It focuses primarily on the AOP framework and discusses several pragmatic, epistemological and regulatory challenges for the use/implementation of non-animal methods. A major strength of the article is the provision of empirical data that makes these problems tangible.

*Most important issues:*

 Methodology: I think the article should report more details on (a) the theoretical sampling approach, (b) how the interviews/focus groups were conducted and (c) on the interpretative analysis of the results (“grounded theory approach” seems a bit general). How did this work in practice? It’s hard to assess the study thoroughly without these information.

 In addition, limitations of the study should be made more explicit in the final section.

 Finally, I would recommend to always make it very clear (in the discussion section and especially in the conclusion) that the respective statements on the challenges are made from the interviewee's perspective. This would avoid the impression that the validity of these statements is uncritically assumed (which would require an independent assessment that goes beyond the scope of the study).

*Minor points:*

 The background section about the AOP framework feels a bit disconnected and should be embedded more clearly into the introduction.

 I think the para starting with “In my own contributions ...“ (p.8.) should be moved to the discussion section.

 Word missing: "Risk assessors and managers work in a wide variety of contexts; they also very different roles within their employment setting, […].“

Is the work clearly and accurately presented and does it cite the current literature?

Yes

If applicable, is the statistical analysis and its interpretation appropriate?

Not applicable

Are all the source data underlying the results available to ensure full reproducibility?

No source data required

Is the study design appropriate and is the work technically sound?

Yes

Are the conclusions drawn adequately supported by the results?

Partly

Are sufficient details of methods and analysis provided to allow replication by others?

Partly

Reviewer Expertise:

Philosophy and social studies of science

I confirm that I have read this submission and believe that I have an appropriate level of expertise to confirm that it is of an acceptable scientific standard, however I have significant reservations, as outlined above.
